# The Effects of Gamification and Oral Self-Care on Oral Hygiene in Children: Systematic Search in App Stores and Evaluation of Apps

**DOI:** 10.2196/16365

**Published:** 2020-07-08

**Authors:** Nino Fijačko, Lucija Gosak, Leona Cilar, Alenka Novšak, Ruth Masterson Creber, Pavel Skok, Gregor Štiglic

**Affiliations:** 1 Faculty of Health Sciences University of Maribor Maribor Slovenia; 2 Dr. Novsak storitve d.o.o. Ljubljana Slovenia; 3 Healthcare Policy and Research Division of Health Informatics Weill Cornell Medicine New York, NY United States; 4 Medical Faculty University of Maribor Maribor Slovenia; 5 Faculty of Electrical Engineering and Computer Science University of Maribor Maribor Slovenia

**Keywords:** mobile health, oral health care, gamification, mobile store, evidence-based dentistry, behavior change technique, Mobile Application Rating Scale user version

## Abstract

**Background:**

Poor oral hygiene is a great public health problem worldwide. Oral health care education is a public health priority as the maintenance of oral hygiene is integral to overall health. Maintaining optimal oral hygiene among children is challenging and can be supported by using relevant motivational approaches.

**Objective:**

The primary aim of this study was to identify mobile smartphone apps that include gamification features focused on motivating children to learn, perform, and maintain optimal oral hygiene.

**Methods:**

We searched six online app stores using four search terms (“oral hygiene game,” “oral hygiene gamification,” “oral hygiene brush game,” and “oral hygiene brush gamification”). We identified gamification features, identified whether apps were consistent with evidence-based dentistry, performed a quality appraisal with the Mobile App Rating Scale user version (uMARS), and quantified behavior scores (Behavior Change score, uMARS score, and Coventry, Aberdeen, and London-Refined [CALO-RE] score) using three different instruments that measure behavior change.

**Results:**

Of 612 potentially relevant apps included in the analysis, 17 met the inclusion criteria. On average, apps included 6.87 (SD 4.18) out of 31 possible gamification features. The most frequently used gamification features were time pressure (16/17, 94%), virtual characters (14/17, 82%), and fantasy (13/17, 76%). The most common oral hygiene evidence-based recommendation was brushing time (2-3 minutes), which was identified in 94% (16/17) of apps. The overall mean uMARS score for app quality was high (4.30, SD 0.36), with good mean subjective quality (3.79, SD 0.71) and perceived impact (3.58, SD 0.44). Sufficient behavior change techniques based on three taxonomies were detected in each app.

**Conclusions:**

The majority of the analyzed oral hygiene apps included gamification features and behavior change techniques to perform and maintain oral hygiene in children. Overall, the apps contained some educational content consistent with evidence-based dentistry and high-quality background for oral self-care in children; however, there is scope for improvement.

## Introduction

Oral diseases caused by oral hygiene are a major problem worldwide. In some countries, more than 80% of school children are affected [1-3]. Oral diseases can cause severe pain and loss of teeth, both of which affect appearance, dietary intake, and consequently the growth and development of children [4,5]. Poor oral hygiene is associated with poor quality of life and increased morbidity and mortality [1-3,6]. The maintenance of oral hygiene or oral self-care, which includes the use of toothbrushes, dental floss, and other interdental aids for healthy teeth, gums, oral soft tissues, palate, tongue, lips, and salivary glands, is important for quality of life, socialization, overall health, and well-being [7-9].

Brushing at least twice a day with toothpaste containing fluoride is considered an important aspect of the prevention and promotion of good brushing habits at an early age to prevent early childhood dental decay [10-12]. Oral health care providers play a key role in promoting oral health care among children [13]. Maintaining optimal oral hygiene can be challenging owing to different factors contributing to the lack of motivation and the need to initiate initial dental assessments between 6 and 12 months of age [14,15].

As children get older, there are new opportunities to use mobile health (mHealth) apps to support pediatric oral hygiene. Gamification is defined as the use of features to increase target behaviors and engagement. The purpose of gamification is to enable users to perform tasks more effectively while making them more enjoyable [16-18]. Gamification features, including badges, levels, and leader boards [17-19], offer novel approaches in dentistry [20]. Gamification has also been included in asthma apps [21] for similar reasons and has been incorporated into other app reviews for fitness apps [22,23], chronic disease management [24], smoking cessation [25], and health promotion [26]. The aim of our study was to identify apps that include gamification features and evidence-based dentistry (EBD) to support the maintenance of oral self-care in children.

## Methods

### Systematic Search Criteria and Selection

All apps were searched in June 2019 across six different smartphone app stores (Google Play Store [27], Apple App Store [28], Windows Phone Store [29], Amazon Appstore [30], BlackBerry World [31], and Samsung Galaxy Apps [32]). Our search strategy had two stages. The first evaluation stage was performed in each app store by using the same four search strings (“oral hygiene game,” “oral hygiene gamification,” “oral hygiene brush game,” and “oral hygiene brush gamification”). The PICO criteria [33] were used to define the search criteria as follows: population (children below 13 years), intervention (free and paid apps that contain enlightenment gamification features and that allow users to be part of the interaction), comparison (app contents), and outcome (suitable apps for learning, performing, and maintaining oral health). Apps were excluded if they were duplicate (from multiple search strings and from multiple official web stores), were games, had a non-English interface, and were not related to the oral health care of children above 13 years. The second stage was performed using smartphones. In this stage, the relevant apps were downloaded and evaluated independently by two reviewers (NF and LG). The same reviewers evaluated the eligibility of the apps against preset criteria. Any issues were resolved by discussion among the other members of the study team. We included all free and paid apps that had two or more gamification features in English. Downloaded apps were analyzed on Samsung Galaxy S8 (Samsung, Seoul, South Korea) running Android 9.0 Pie (Google Inc, Mountain View, California, USA) for Android apps and iPhone 7 running iOS 12.3.1 (Apple Inc, Cupertino, California, USA) for iOS apps. If the same app was found for both Android and iOS, we reviewed the app available on Android, as the Google Play Store provides more information about each app than that available on the Apple App Store [34].

### Measures and Rating Tools

All ratings and rankings were conducted individually and independently by authors (NF, LG, AN, and LC) with experience, knowledge, and skills in the field of mHealth and experience in different fields of health care (nursing, bioinformatics, and dentistry) to support the synthesis of the search results.

#### Rating Tool for Gamification Features

Two authors (LC and NF) identified gamification features by using the modified taxonomy of 31 gamification components (Multimedia Appendix 1) [19,35]. The scoring for gamification features was 1 point for the full implementation of gamification features, 0.5 points for partial implementation, and 0 points if the feature was not implemented.

#### Rating Tool for Oral Hygiene-Related Content Based on Dentistry Evidence

We evaluated EBD in each app using criteria and scored them according to the following four groups: (1) preparing to brush (two items); (2) before brushing (one item); (3) brushing (four items); and (4) after brushing (three items) (Multimedia Appendix 2) [36-40].

#### Mobile App Rating Scale User Version and Quantifying Behavior Change

Based on the Mobile App Rating Scale user version (uMARS) [41], we performed quality appraisal of apps. Originally, the MARS tool was developed with the aim that researchers could use it to determine whether apps satisfied certain quality criteria instead of relying on a subjective five-star rating system [41,42]. The uMARS is a simplified version of MARS [42], which allows multidimensional measurements (sections A-F) of performance indicators (section A), functionality (section B), esthetics (section C), quality of information (section D), subjective quality of the app (section E), and perceived impact (section F). The uMARS tool was developed to eliminate the need for trained professionals, and according to a study by Stoyanov et al, it has good internal consistency (α=.09), and high reliability [41]. All items were rated on a 5-point Likert scale from 1 (inadequate) to 5 (excellent) and were already used in a similar study [43]. Behavior change was also rated and ranked according to three taxonomies (Behavior Change score [44], uMARS score [41], and Coventry, Aberdeen, and London-Refined [CALO-RE] score [45]) related to behavior change (Multimedia Appendix 3 and Multimedia Appendix 4). Similar to the rating tool for gamification features, the scoring system allowed 0, 0.5, or 1 point according to implementation. The exception was the uMARS score where items were scored based on a Likert scale from 1 (strongly disagree) to 5 (strongly agree).

### Data Analysis

All data analyses and visualizations were prepared using Microsoft Office Professional 2016 (Microsoft Excel 2016; Microsoft Corp, Redmond, Washington, USA) and IBM SPSS Statistics version 25 for Windows (IBM Corp, Armonk, New York, USA). Intraclass correlation coefficients (ICCs) were calculated to provide results on consistency among observational ratings provided by multiple raters [46].

## Results

### Descriptive Characteristics

**Figure 1:**
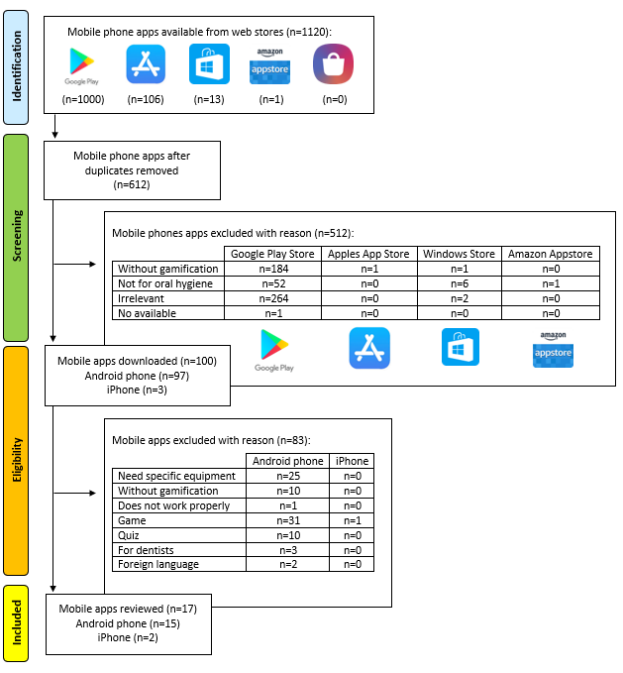
PRISMA flow diagram of the selection of the included apps.

We identified 612 potentially relevant apps, and only 17 (3%) met the inclusion criteria. The Preferred Reporting Items for Systematic Reviews and Meta-Analyses (PRISMA) flow diagram [47] presents the process of scanning the apps using the inclusion and exclusion criteria. The two largest groups of apps that were excluded in the PRISMA diagram (Figure 1) were apps that required specific equipment or accessories [48] and apps that included gamification but did not include any interaction with the user [49].

The vast majority of apps were free to download (11/17, 65%) and were available in both the Google Play Store and Apple App Store (14/17, 82%). Most apps did not require registration (14/17, 82%) and were updated in the last 2 years (13/17, 76%). Apps were developed in multiple countries, including the United States (6/17, 35%), Japan (2/17, 12%), Australia (1/17, 6%), Germany (1/17, 6%), England (1/17, 6%), Lithuania (1/17, 6%), North Korea (1/17, 6%), South Korea (1/17, 6%), Russia (1/17, 6%), Sweden (1/17, 6%), and Ukraine (1/17, 6%). Apps were developed by small and medium-size enterprises (14/17, 82%), health care–related agencies (2/17, 12%), and an individual (1/17, 6%).

Apps were targeted at different age groups of children. Among the 17 apps, 1 (6%) app was for children aged 5 years or less, 7 (41%) were for children aged 5 to 8 years (41%), 2 (12%) were for children aged 6 to 12 years, and 7 (41%) did not provide information about the target age of children but had a Pan European Game Information 3 (PEGI) certificate. 

Table 1 presents all the relevant apps and their basic characteristics [50-80]. We assessed the subjective quality of the apps by answering the following question included in the uMARS framework: “What is your overall (star) rating of the app?” The quality of the apps was rated on a 5-point Likert scale from 1 (the worst app I have used) to 5 (the best app I have used). The mean app score was 3.79 (SD 0.69). The lowest score was 3, and the highest score was 5. We compared the results with the ratings of users in the smartphone app stores. If the app ratings were available on both smartphone platforms (Android and iOS), we used the mean score. The mean user rating was 4.26 (SD 0.29) (Android: 4.21, SD 0.34; iOS: 4.33, SD 0.54), and the mean number of raters was 4981.79 (SD 12023.77) for Android apps and 359.62 (SD 934.17) for iOS apps. Based on a *t* test, there was a statistically significant difference between uMARS estimates (mean 3.85, SD 0.16) and user ratings (mean 4.26, SD 0.30; t_32_=−2.27; *P*=.03).

**Table 1:** Description of the included apps.

Full app name	Smartphone platform	Rating in the form of stars (number of raters)	
		Google Play Store	Apple App Store
Brush DJ [50,51]	Android & iOS	4.2 (1363)	4.7 (948)
Brush Hero [52,53]	Android & iOS	—^a^	4.2 (24)
Brushing Hero [54,55]	Android & iOS	4.5 (371)	4.2 (‎24)
Brush Monster [56,57]	Android & iOS	4.4 (199)	5.0 (2)
Brush My Teeth [58,59]	Android & iOS	4.6 (5)	—
Brush'n'Save [60,61]	Android & iOS	4.0 (87)	—
Brush Teeth with The Wiggles [62,63]	Android & iOS	4.3 (132)	4.8 (‎8)
Brush Up [64,65]	Android & iOS	3.8 (736)	4.1 (64)
Chomper Chums [66,67]	Android & iOS	4.1 (355)	5.0 (7)
Disney Magic Timer by Oral-B [68,69]	Android & iOS	4.0 (36,434)	3.9 (3348)
Mimizavr Clean Teeth [70,71]	Android & iOS	4.0 (14)	—
MyTeeth [72,73]	Android & iOS	5 (1)	3.0 (9)
My Virtual Tooth - Virtual Pet [74,75]	Android & iOS	4.3 (29,945)	4.3 (197)
Timo Kids Routine Timer [76,77]	Android & iOS	4.1 (100)	4.0 (1)
Tooth Hero [78]	Android	3.7 (3)	—
Toothsavers Brushing Game [79]	iOS	—	4.6 (8)
Toothy: Toothbrush Timer [80]	iOS	—	4.5 (34)

^a^Not available.

### Gamification Features

**Figure 2:**
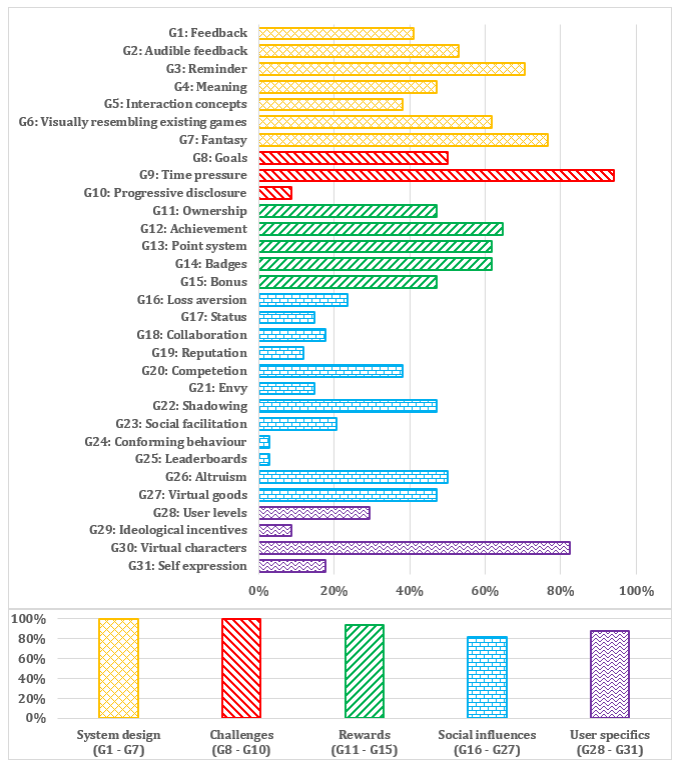
Gamification component categories of gamification features.

On average, apps included 6.87 (SD 4.18) out of 31 possible gamification features. The most frequent gamification features were time pressure (16/17, 94%), virtual characters (14/17, 82%), and fantasy (13/17, 76%). The less frequently represented gamification features were conforming behavior and leaderboards (each 0.5/17, 3%) (Figure 2).

We identified all apps based on the gamification features (17/17, 100%) from the system design and challenges part of gamification component categories (Figure 2).

**Figure 3:**
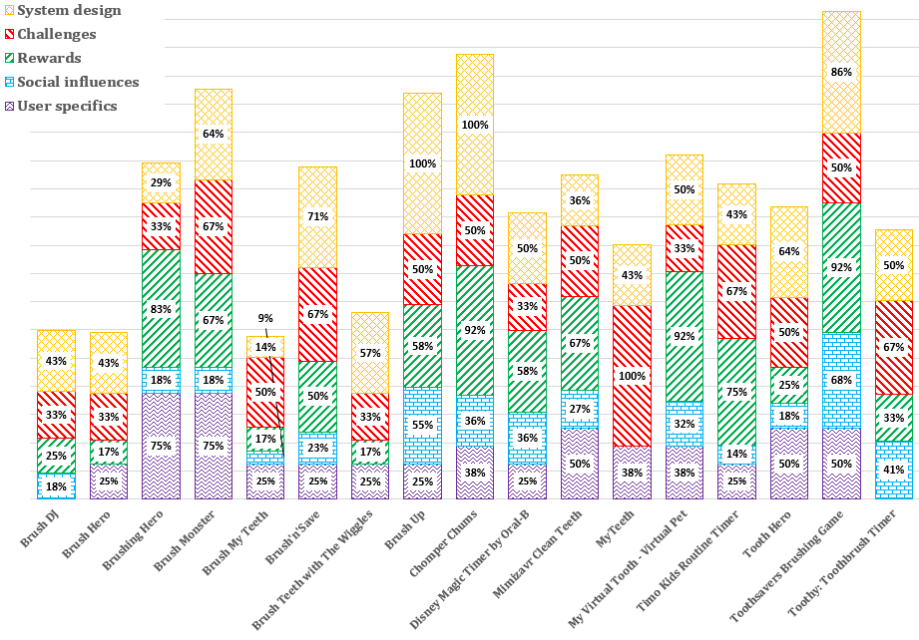
Percentage of gamification component categories for each app.

Apps in which we identified the most gamification component categories of gamification features were Toothsavers Brushing Game [79], Chomper Chums [66,67], Brush Monster [56,57], and Brush Up [64,65] (Figure 3).

### Oral Hygiene-Related Content Based on Dentistry Evidence

**Figure 4:**
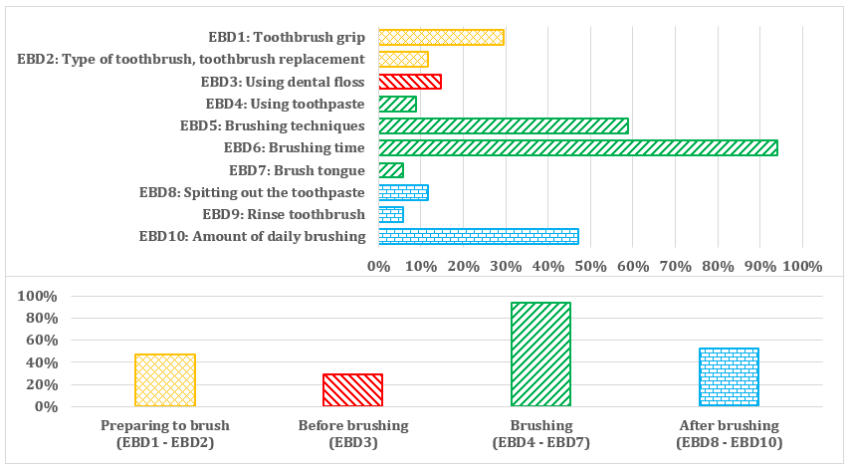
Group categories of evidence-based dentistry (EBD) oral hygiene-related content.

On average, we identified 4.9 (SD 4.98) oral hygiene-related contents in 10 groups based on the level of EBD (EDB1-EDB10). The most common EBD content focused on brushing time (Figure 4) from the EBD group category “Brushing.” Most apps (14/17, 82%) had brushing time set to 2 minutes whereas the app Brushing Hero [54,55] had it set to 1 minute. Only two apps (MyTeeth [72,73] and Brush Up [64,65]) had it set to 3 minutes.

**Figure 5:**
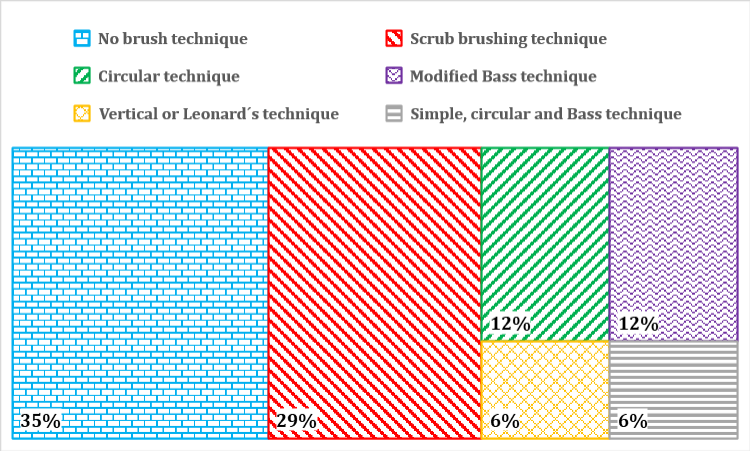
Brushing techniques used in the apps.

The most common brushing technique was the scrub brush technique (5/17, 29%). One app (MyTeeth [72,73]) allowed the user to choose among different brushing techniques (scrub brush technique; circular technique; Bass technique; and vertical technique from red [gums] to white [teeth], or inside, chewing surface, and outside or chewing surface, outside, and inside). Over one-third of the apps did not mention any EBD brushing technique (Figure 5).

**Figure 6:**
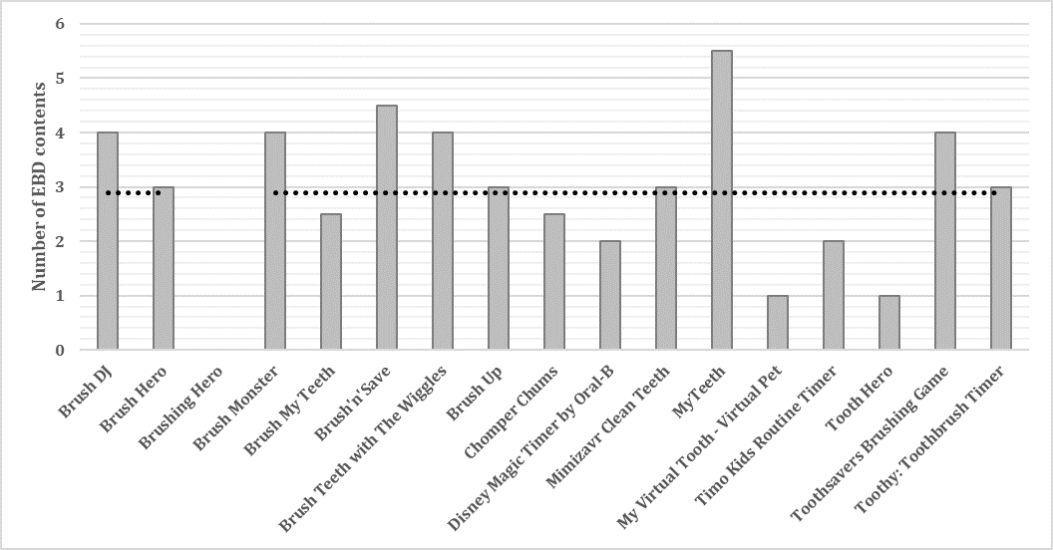
Number of evidence-based dentistry (EBD) contents in the apps.

On average, the apps included almost 3 out of 10 EBD contents (Figure 6). The MyTeeth app included the most EBD contents.

### uMARS App Quality Scores and Oral Hygiene Care Behavior Change Techniques

The total mean score for the section “app quality” was 4.30 (SD 0.36), which is considered a good result. The ICC among app reviewers was high for uMARS ratings (ICC=0.80; 95% CI 0.63-0.92). Toothsavers Brushing Game [79] had the highest score in each section of the uMARS (Table 2).

**Table 2:** App scores for each section of the Mobile App Rating Scale user version.

Full app name	uMARS^a^ section						
	Engagement	Functionality	Esthetics	Information	App quality	App subjective quality	Perceived impact
Brush DJ	3.5	4.5	3.5	4.1	3.9	3.5	3.8
Brush Hero	3.9	3.1	4.7	—^b^	3.9	2.5	2.9
Brushing Hero	4.4	4.8	4.7	—	4.6	4.0	3.3
Brush Monster	5.0	4.8	5.0	4.4	4.8	4.9	4.0
Brush My Teeth	3.7	4.4	4.3	3.1	3.9	3.3	3.2
Brush'n'Save	3.8	5.0	2.5	4.3	3.8	3.9	3.6
Brush Teeth with The Wiggles	4.4	4.8	4.0	—	4.4	3.5	3.2
Brush Up	4.3	4.6	4.3	4.5	4.4	5.0	4.2
Chomper Chums	4.7	4.6	4.2	4.3	4.4	4.1	4.1
Disney Magic Timer by Oral-B	4.3	4.8	4.2	3.0	4.3	3.1	3.2
Mimizavr Clean Teeth	4.5	5.0	5.0	—	4.8	4.3	3.5
MyTeeth	3.2	4.6	4.0	—	3.9	3.3	3.7
My Virtual Tooth - Virtual Pet	4.4	4.6	4.0	—	4.3	3.6	3.1
Timo Kids Routine Timer	4.0	4.9	4.2	—	4.4	3.1	3.6
Tooth Hero	4.4	4.0	4.0	2.5	4.5	4.0	3.7
Toothsavers Brushing Game	4.9	4.9	4.8	4.8	4.9	5.0	4.5
Toothy: Toothbrush Timer	3.1	4.4	4.2	4.0	3.9	3.4	3.3
Mean score (SD)	4.15 (0.55)	4.58 (0.46)	4.21 (0.60)	3.90 (0.76)	4.30 (0.36)	3.79 (0.71)	3.58 (0.44)
ICC^c,d^	0.85	0.71	0.78	0.98	0.56	0.88	0.84

^a^uMARS: Mobile App Rating Scale user version.

^b^Not available; in 7 out of 17 (41%) apps the “information” quality section could not be assessed because data were not included.

^c^ICC: intraclass correlation coefficient.

^d^A random effects average measures model with absolute agreement was used for calculated ICCs among two sets of ratings.

We also evaluated apps based on “subjective quality,” where the mean estimate was good (mean 3.79, SD 0.71) and on “perceived impact,” where the mean estimate was also good (mean 3.58, SD 0.44) (Table 2).

**Figure 7:**
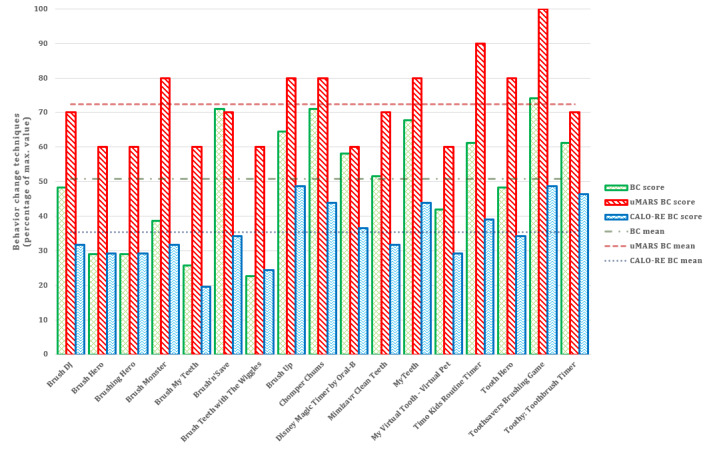
Number of oral hygiene care behavior change techniques. BC: Behavior Change; CALO-RE: Coventry, Aberdeen, and London-Refined; uMARS: Mobile App Rating Scale user version.

Results from three behavior change scores across three instruments indicated that Brush Up [64,65], Toothsavers Brushing Game [79], and Chomper Chums [66,67] included the most strategies targeting behavior change (Figure 7). The most common oral hygiene care behavior change techniques were “prompt intention formation” and “model or demonstrate the behavior.”

## Discussion

### Gamification Features

The primary objective of this study was to identify apps that include gamification features focused on motivating children for behavior change to maintain oral self-care and optimal oral hygiene. The study results showed that most of the oral hygiene apps included a relevant number of gamification features and behavior change techniques to perform and maintain oral hygiene in children. Additionally, many apps contained EBD-supported educational content and achieved relatively high scores in app quality ratings. The most commonly identified gamification features were “time pressure” and “audible feedback” in the form of songs that help children to practice oral health care correctly. We compared our gamification results with a study conducted in a pediatric population [81] and found similar results with a focus on “feedback” and “goals” versus “information provision (general)” and “goal setting (behavior).” The authors also concluded that using apps for oral health care can improve motivation for oral self-care in children. Moreover, recent research found that knowledge provision increased motivation in children and that gamification might improve engagement with apps [49].

### Apps Content Based on Dentistry Evidence

Our second aim was to determine what should be included in EBD health care apps. A small proportion of apps included EBD and the most frequently included EBD content was brushing time. Oral health care professionals generally recommend at least 2 or 3 minutes for brushing teeth. A study from 2019 showed increased duration of toothbrushing when using the Brush Up app [81].

The brushing technique is important for optimal oral self-care. It is recommended that children use a modified Bass technique because of efficient plaque removal compared with other toothbrushing techniques [82,83]. The scrub brushing technique was the most frequently suggested in the reviewed apps. The scrub brushing technique is the easiest to program into an app and the most commonly used technique in the general population [82,84].

Children should perform oral hygiene activities more than two times a day, and yet, less than half of the apps included proper EBD warnings or reminders for users to brush their teeth at least twice a day. We also found that only a few apps emphasized brushing with a proper toothbrush and toothpaste quantity, as supported by the literature to reduce the incidence of decay [82,85]. The recommended “pea-sized amount,” which represents approximately 1000 parts per million (ppm) fluoride, is generally recommended, yet only two apps visualized the correct quantity of toothpaste.

Another important aspect of oral hygiene is the use of a soft or extra-soft toothbrush for safe oral health care [86]. Older brushes lose their plaque removal ability [87,88] and are often contaminated with microbes [89]. Guidelines recommend replacing toothbrushes when they show signs of bristle splaying tear. In addition to oral self-care, less than 20% of apps included the use of dental floss or other interdental brushing approaches. None of the apps promoted the EBD recommendation to first floss and then brush [90,91].

Although the usage of apps to monitor and manage health is increasing, it is difficult to find information about the sources used in the development of the apps [92]. In addition, many app developers focus on the usefulness and ease of use of the app instead of the quality of the content included in the app.

### App Quality and Behavior Change Techniques

Our results showed that average users tended to give more positive feedback to apps based on functionality [93] and popularity [94], and there was less focus on usability or quality, which can potentially represent dangerous health information for future users. Therefore, researchers often rely on more complex evaluation tools like the uMARS [95] to obtain a better estimation of app quality. Overall, the Toothsavers Brushing Game [79] had the highest quality score according to the uMARS. Additionally, Toothsavers Brushing Game [79] had the highest scores in all categories of gamification components, EBD oral hygiene-related content, and all three behavior change technique taxonomies, which is consistent with our results. Brush Monster [56,57] and Chomper Chums [66,67] were also visually well designed, included EBD content, and did not have any specific technical problems during their use.

One of the most important behaviors in oral hygiene care change techniques is user interaction with the app [96]. Our results showed that one app included learning of oral self-care with virtual model teeth and enabled the user to perform self-based oral health care interaction in the app. In the apps Brushing Hero [54,55] and Brush Monster [57,58], augmented reality is added to the self-based oral health care interaction, where the user is a role model. Other apps use one or more different teaching approaches for oral self-care in the form of virtual teeth or a virtual tooth, gradually revealing a picture, virtual model teeth, video, and games.

We also must consider the risk of using apps as an intervention for oral hygiene care in children, as the youngest users seem to be most highly affected by and at risk for behavioral addictions [97]. The pattern of smartphone abuse is greatest among young people, and problematic use is linked to self-esteem, impulsivity, self-identity, and self-image. Problematic use is also associated with sleep disturbance, anxiety, stress, and, to a lesser extent, depression [98], as well as impaired psychological well-being, impaired parent and school relationships, and additional behavioral problems [99]. As interventions using apps or games for increasing oral hygiene care quality can be successful, judicious use is advised. Our observation is that using apps for learning, performing, and maintaining oral self-care requires less than 10 minutes every day, and this is much lower than the average time spent on a smartphone.

### Observation of Apps and Tips for Future Oral Health Care App Developers

Our study provides app reviewers and developers of oral self-care with structured information on how future apps should be implemented and which EBD content should be included. Apps for supporting oral self-care in oral hygiene should have a combination of gamification features and EBD. Collaboration with oral health care professionals (eg, oral health care organizations and dentists) can improve the content provided in the app and bring EBD self-care information to targeted end users.

We found the following two secondary app payment strategies: “freemium” (eg, in-app purchases) and “free trial” (eg, free to premium version). Three apps had a trial version and required payment over time (Toothy: Toothbrush Timer [80], €1.32/month [US $1.42/month]; Brush Up [64,65], €1.11/month [US $1.20/month] or buy €11.14 [US $12.02]; My Virtual Tooth-Virtual Pet [74,75], €5.49 [US $5.92]). In-app purchases in different forms were offered in five apps, with a minimum price of €0.99 (US $1.07) and maximum price of €11.14 (US $12.02) per item. To increase usage, many mobile game companies provide ‘‘freemium” services, which cost the player nothing for basic usage but need payment with real money for advanced functionality or virtual goods [100]. Liu et al found that the freemium strategy is positively associated with increased sales of paid apps [101]. The most popular in-app purchases were in the form of getting virtual goods (ie, coins), disabling in-app advertisements, getting virtual characters (eg, avatars), and achieving virtual goals (eg, levels). Only three apps in the smartphone app stores provided information that the apps contain advertisements and offer in-app purchases. Affective states, such as playfulness, seem to be one of the most influential factors positively affecting consumers’ intention to pay for mobile game services [102]. Additionally, “stickiness,” that is, the trait of a game that engages users, is found to greatly and positively influence in-app purchases [103].

Positive effective states associated with in-game rewards seem to help the user to play the game better and can enhance both perceived value and customer loyalty [104]. Many smartphone games that provide “freemium“ services are intended to be used by children. Hence, it seems that smartphone game service providers try to use the affective states of children to increase their revenue. This is especially concerning since the preadolescent (and adolescent) brain is still very much developing, especially in terms of reasoning and anticipating what will happen in the future [105]. Only one app (Disney Magic Timer by Oral-B [68,69]) alerted users that real money is needed to make in-app purchases; therefore, parental supervision is recommended. Google Play Store and Apple App Store provide users the opportunity to get a refund of the money paid but within a limited period.

### Limitations

As with any review of commercially available tools in app stores, there is time sensitivity of the results because apps are being added and removed from app stores daily. Given that caveat, this review is meant to provide an overview of the state of apps available in this field, with less emphasis on individual apps. Another limitation of this study is that it focused only on features that were freely available in the apps. As such, we may have missed gamification features, EBD contents, and other features in the apps. We also excluded apps that required subscription over time. The last limitation in this study is that we did not make any in-app purchases in paid apps, so we could not present additional results for in-app purchases.

### Future Research

Future work should evaluate one of the highest scoring apps (eg, Toothsavers Brushing Game and Chomper Chums) in a randomized controlled trial to evaluate oral hygiene outcomes and motivation for learning, performing, and maintaining oral self-care among children.

### Conclusions

In this systematic review, we did not find any app that had all the segments for learning, performing, and maintaining proper oral health care. The Toothsavers Brushing Game app had the highest scores, and if future updates introduce more EBD content, it could be the most appropriate app for learning, performing, and maintaining good oral health care in children. Gamification features with EBD have good potential as new approaches for health care providers to change behavior in the form of learning, performing, and maintaining proper oral hygiene with EBD in the clinical environment.

## Appendix

Multimedia Appendix 1 Exemplary gamification features rating criteria for oral hygiene apps.

Multimedia Appendix 2 Oral self-care app segments and evidence-based dentistry.

Multimedia Appendix 3 Exemplary oral health care behavior change techniques according to the Behavior Change score.

Multimedia Appendix 4 Exemplary oral health care behavior change techniques according to the Coventry, Aberdeen, and London-Refined behavior change score.

## Abbreviations

EBD: evidence-based dentistry
ICC: intraclass correlation coefficient
PRISMA: Preferred Reporting Items for Systematic Reviews and Meta-Analyses
uMARS: Mobile Application Rating Scale user version
