# Supplementary material for: The Effects of Gamification and Oral Self-Care on Oral Hygiene in Children: Systematic Search in App Stores and Evaluation of Apps
Source: JMIR Mhealth Uhealth. 2020 Jul 8;8(7):e16365. doi: 10.2196/16365 (PMC7381071; doi:10.2196/16365)
Supplement: Multimedia Appendix 1 [file mhealth_v8i7e16365_app1.docx]

**Supp 1: Exemplary gamification features rating criteria for oral hygiene apps**

| GCC | ID | Gamification features | App archetypes in oral hygiene apps |
| --- | --- | --- | --- |
| System design | G1 | Feedback | Comparing oral hygiene related goals to recorded oral hygiene data and provide feedback. |
|  | G2 | Audible feedback | Any kind of audible feedback that is related to certain results or behaviors. |
|  | G3 | Reminder | Reminder for oral hygiene. |
|  | G4 | Meaning | Providing information on oral hygiene such as causes, triggers, symptoms and FAQs. |
|  | G5 | Interaction concepts | Providing an aesthetic user interface combined with a high degree of usability. |
|  | G6 | Visually resembling existing games | Resembling common games in a visual way. |
|  | G7 | Fantasy | Using fantasy elements such as fairy tales, speaking animals etc. |
| Challenges | G8 | Goals | Personalized tasks and challenges set to increase oral hygiene control. |
|  | G9 | Time pressure | Creating time pressure through timers or countdowns to incentivize certain behaviors. |
|  | G10 | Progressive disclosure | Increasing the difficulty of oral hygiene related tasks continuously. |
| Rewards | G11 | Ownership | Creating a feeling of ownership through functions such as quiz scores or personalized avatars. |
|  | G12 | Achievement | Rewarding accomplished oral hygiene goals, e.g. with virtual gifts, new app features, or high score rankings. |
|  | G13 | Point system | Measuring and comparing the user’s oral hygiene related accomplishments with numerical values. |
|  | G14 | Badges | Awarding batches as a sign for oral hygiene goal accomplishments. |
|  | G15 | Bonus | Granting extra rewards for accomplishing a series of oral hygiene related goals. |
| Social influences | G16 | Loss aversion | Punishing the user if planned oral hygiene related behavior is not performed. |
|  | G17 | Status | Providing the possibility for social comparison with other users through social network features. |
|  | G18 | Collaboration | Providing the possibility for social interaction with physicians or other users. |
|  | G19 | Reputation | Providing peer networks for social interaction. |
|  | G20 | Competition | Enabling users to challenge each other in reaching oral hygiene related goals. |
|  | G21 | Envy | Providing mechanisms of social comparison that lead to personal envy. |
|  | G22 | Shadowing | Giving users the ability to compare their results with previous scores such as number of brushing in one day. |
|  | G23 | Social facilitation | Goal-related interactions with oral hygienist or dentist (also caregivers and parents). |
|  | G24 | Conforming behavior | Providing peer networks, which enable peer pressure to increase or decrease oral hygiene related actions. |
|  | G25 | Leaderboards | Tracking and ranking of oral hygiene related accomplishments with leaderboards. |
|  | G26 | Altruism | Enabling users to give away virtual gifts. |
|  | G27 | Virtual goods | Enabling users to buy or trade virtual goods. |
| User specifics | G28 | User levels | Indicating the user’s proficiency through different levels. |
|  | G29 | Ideological incentives | Information about controlled and uncontrolled oral hygiene especially regarding the consequences for a patient’s quality of life and potential health risks |
|  | G30 | Virtual character | The user is represented by a virtual character. |
|  | G31 | Self-expression | The user is given the ability for self-expression through customizable functions. |

GCC=Gamification component categories of gamification features
