# Supplementary material for: The Effects of Gamification and Oral Self-Care on Oral Hygiene in Children: Systematic Search in App Stores and Evaluation of Apps
Source: JMIR Mhealth Uhealth. 2020 Jul 8;8(7):e16365. doi: 10.2196/16365 (PMC7381071; doi:10.2196/16365)
Supplement: Multimedia Appendix 2 [file mhealth_v8i7e16365_app2.docx]

**Supp 2: Oral self-care app segments and evidence-based dentistry**

| **Group categories of toothbrushing related content** | **Archetypes in oral health content in apps** | **Benefits to oral hygiene** | **Points ranking based on EDB** | **Type of evidence-based dentistry study** | **Quartile of evidence-based dentistry (journal, impact factor, year)** |
| --- | --- | --- | --- | --- | --- |
| Preparing to brush | EBD1: Determine toothbrush grip. | In research oblique, distal oblique and power grips were most efficient (plaque reduction up to 70%) [36]. | Distal oblique, oblique, power, precision and spoon toothbrush grip (1 point). | The power analysis. | Q2 (European Archives of Paediatric Dentistry, 0.83, 2018). |
|  | EBD2: Determine bristle stiffness of toothbrush, detecting information about toothbrush replacement. | Soft and extra-soft toothbrushes tend to be safer [86]. Older brushes lose their plaque removal ability [87]. | Extra‐soft and soft toothbrushes or replacement toothbrush after 3 mounts  (1 point), medium and hard toothbrushes or replacement toothbrush without specific time (0.5 point). | A systematic review, investigate study. | Q2 (International Dental Journal, 1.69, 2018), Q2 (International journal of dental hygiene, 0.80, 2018). |
| Before brushing | EBD3: Determine usage of dental floss. | Dental flossing followed by brushing provides better oral hygiene [90,91]. | Using dental flossing before brushing  (1 point), using dental flossing anytime (0.5 point). | A randomized controlled clinical trial. | Q1 (Journal of Periodontology, 1.64, 2018). |
| Brushing | EBD4: Determine usage of toothpaste in form of type and amount. | Fluoride plays an important role in preventing tooth decay. They recommend toothbrush with 1000 to 1500 ppm of fluoride [12]. | Using toothpaste which fluoride in amount of 1000-1500 ppm (1 point), using toothpaste with fluoride or using toothpaste in amount of 1000-1500 ppm (0.5 point). | Non-systematic review. | Q1 (Pediatric Clinics of North America, 0.37, 2018). |
|  | EBD5: Determine brushing techniques. | In younger children is advised the horizontal technique [37,82]. | Horizontal scrub, Fones technique, Modified Bass and others (1 point). | Randomized clinical studies. | Q2 (Oral Health & Preventive Dentistry, 0.41, 2018), Q1 (British Dental Journal, 1.48, 2018). |
|  | EBD6: Determine brushing time. | With increased brushing time, we have an increased plaque removal [36]. | Two or more minutes (>2.5 min) (1 point). | The power analysis. | Q2 (European Archives of Paediatric Dentistry, 0.83, 2018). |
|  | EBD7: Determine cleaning tongue. | Tongue cleaning reduced the bacteria in tongue coating [38]. | Using tongue cleaner (1 point). | Randomized examiner-blind crossover study. | Q1 (BMC Oral Health, 1.41, 2018). |
| After brushgin | EBD8: Determine of spitting out the toothpaste. | The new guidelines recommend spitting and avoid excessive rinsing. Rinsing with water after brushing with fluoride toothpaste can reduce the benefit of fluoride toothpaste [39]. | Spitting out the toothpaste (1 point). | Exploration of the available evidence. | Q1 (British Dental Journal, 1.48, 2018). |
|  | EBD9: Determine rinsing toothbrush after brushing. | After brushing rinse, the toothbrush with tap water to remove any toothpaste and debris [40]. | Rinsing toothbrush after brushing (1 point). | Guidelines. | Q1 (Journal of American Dental Association, 2.572, 2018). |
|  | EBD10: Amount of daily brushing. | For school children, tooth wash twice daily or more is recommended for proper oral hygiene [11]. | Twice a day or more (1 point). | Longitudinal study. | Q2 (International dental journal, 1.628, 2018). |
