# Supplementary material for: The Effects of Gamification and Oral Self-Care on Oral Hygiene in Children: Systematic Search in App Stores and Evaluation of Apps
Source: JMIR Mhealth Uhealth. 2020 Jul 8;8(7):e16365. doi: 10.2196/16365 (PMC7381071; doi:10.2196/16365)
Supplement: Multimedia Appendix 3 [file mhealth_v8i7e16365_app3.docx]

**Supp 3: Exemplary behavior of oral health care change techniques for BC score**

| ID | Behavior oral healthcare change techniques | Application archetypes in oral hygiene apps |
| --- | --- | --- |
|  | Provide information about  behavior health link | General information about oral hygiene such as oral diseases, tooth decay and others. |
|  | Provide information on  consequences | Information about controlled and uncontrolled oral hygiene particularly regarding the consequences for a children’s quality of life and potential health risks. |
|  | Provide information about  others’ approval | The app user can interact with dentists and/or other app users through messages and social network features. |
|  | Prompt intention formation | Encourage user to engage in promoting behaviors for implementation oral hygiene. |
|  | Prompt barrier identification | Determine barriers to oral hygiene and provide relevant information and strategies of tackling. |
|  | Provide general  encouragement | Reward or praise the user for the regular and proper implementation of oral hygiene and for engaging with the app. |
|  | Set graded tasks | Personalized task and challenges set to improve the performance of oral hygiene. |
|  | Provide instruction | Instructing the user how to engage with the app and/or oral hygiene behavior such instructions for proper oral hygiene, diary. |
|  | Model or demonstrate the  behavior | Video tutorials, animations, or visualized descriptions of the instructions described in B8. |
|  | Prompt specific goal setting | Setting specific behavior goals to enhance oral hygiene. |
|  | Prompt review of behavioral  goals | Display tracked oral hygiene data and behavior goals (e.g. set in B7). |
|  | Prompt self-monitoring of  behavior | Enabling user to track data for oral hygiene (brushing time, brushing direction, etc.). |
|  | Provide feedback on  performance | Compare oral hygiene control related goals to recorded oral hygiene data and provide feedback. |
|  | Provide contingent rewards | Rewarding and/or praising users for accomplishing health tasks. |
|  | Teach to use prompts or cues | Remind users of environmental stimuli which are related to oral health (e.g. unhealthy diet, smoking, alcohol). |
|  | Agree on behavioral contract | Setting up an adjustable action plan agreed on with a dentist. |
|  | Prompt practice | Notifications for performing oral hygiene (reminder). |
|  | Use follow-up prompts | Oral hygiene notifications, but by messages and/or mails after app is no longer used. |
|  | Provide opportunities for  social comparison | Provide the possibility for social comparison with other users  through social network features. |
|  | Plan social support or social  change | Provide peer networks through social network features and  encourage users to use them for social support |
|  | Prompt identification as a role model | Empower the user to serve as a role model for regular oral hygiene. |
|  | Prompt self-talk | Diaries and journal entries about oral hygiene. |
|  | Relapse prevention | Help users to avoid possibly risky situations for occurrence of oral diseases. |
|  | Stress management | Ease user’s stress through relaxation exercises. |
|  | Motivational interviewing | Encourage self-motivating statements in general or in relation to oral hygiene. |
|  | Time Management | Adjustable reminders for oral hygiene tasks. |
