# Supplementary material for: The Effects of Gamification and Oral Self-Care on Oral Hygiene in Children: Systematic Search in App Stores and Evaluation of Apps
Source: JMIR Mhealth Uhealth. 2020 Jul 8;8(7):e16365. doi: 10.2196/16365 (PMC7381071; doi:10.2196/16365)
Supplement: Multimedia Appendix 4 [file mhealth_v8i7e16365_app4.docx]

**Supp 4: Exemplary behavior of oral health care change techniques for CALO-RE BC score**

| ID | Behavior change techniques | App archetypes in oral hygiene apps |
| --- | --- | --- |
| 1. | Information provision (general) | Provision of general information about oral hygiene health and its possible outcomes. |
| 2. | Information provision (to the individual) | Provision of specific information relevant to the individual about oral hygiene health for his age, oral health status, its possible outcomes, consequences and benefits. |
| 3. | Information provision (others’ approval) | Provision of information about what others might think of their oral hygiene habits. |
| 4. | Information provision (others’ behaviour) | Provision of information concerning what others typically do with respect to oral hygiene. |
| 5. | Goal setting (behaviour) | Encouragement to begin or maintain behaviour change. It does not involve precise planning for the behaviour sequence or performance. A goal may be to “brush teeth twice daily from next week”. |
| 6. | Goal setting (outcome) | The individual is encouraged to set general goals achievable through performance of the behaviour, but distinct from the behaviour itself. A consultant might highlight good periodontal health or decrease in halitosis as achievable goals derived from regular oral hygiene. |
| 7. | Action planning | Detailed plans are made including when (e.g. frequency) and where (e.g. in what situation) to act. Such plans are often expressed in “if–then” formats. For example, a child might plan this “if I win this game, then I will brush twice a day”. |
| 8. | Identifying barriers/problem resolution | After formation of a clear plan, individuals are tasked with identifying possible barriers to performance and solutions to the possible problems. Barriers may be cognitive, emotional, social and/or physical. For instance, “I don’t get time to brush properly because of school rush,—therefore, I will go to sleep earlier every day”. |
| 9. | Setting graded tasks | The target behaviour is broken into smaller, more manageable tasks, allowing successful progression in small increments. For instance, writing down a sequence of small steps to accomplish the overall behaviour over time. |
| 10. | Review of behavioral goals | An opportunity for the individual to review the successful accomplishment of previously set goals. |
| 11. | Review of outcome goals | A review or analysis of the attainment of previously set outcome goals, and an opportunity to revise plans to attain them. |
| 12. | Effort or progress contingent rewards | The person uses rewards or praise for attempts at achieving the goal. |
| 13. | Successful behaviour contingent rewards | Providing rewards for successful performance of the target behaviour. |
| 14. | Shaping | Graded contingent rewards for movements towards completion of the target behaviour. |
| 15. | Generalization of target behaviour | After successful performance of a behaviour in one situation, the person is encouraged to find opportunities to try it in other situations, ensuring the behaviour does not become situation specific. |
| 16. | Self-monitoring of behaviour | The person is asked to keep a detailed record about brushing teeth and use as a means to change or modify behaviour. |
| 17. | Self-monitoring of behavioural outcome | As point 16, but focusing on measurable outcomes of the behaviour (e.g. plaque reduction). |
| 18. | Focus on past success | Using reflections on successful past experience with brushing teeth as a means to increase motivation to be active in future. |
| 19. | Provide feedback on performance | Provision of feedback to an individual regarding a recent oral hygiene success with the aim of increasing motivation to be more active in future. |
| 20. | Informing when and where to perform the behaviour | Offering advice and ideas on when and where an oral hygiene could be performed. |
| 21. | Instruction on how to perform the behaviour | Instructing a person exactly how to effectively perform a behaviour. |
| 22. | Demonstration of behaviour | Showing the person how to perform brushing teeth, through physical or visual means. |
| 23. | Training to use prompts | Instruction on use of cues to help remind people to perform a behaviour. For instance, encouraging toothbrushing use and increasing frequency of brushing or mobile phone alerts to remind them. |
| 24. | Environmental restructuring | The person is prompted to make changes to their environment to facilitate changes in behaviour. |
| 25. | Agreement of behavioural contract | A written agreement between the individual and the practitioner with respect to behaviour change. |
| 26. | Prompt practice | Reminding the person to brush your teeth and repeat the behaviour or situations that lead to the behaviour. This helps reinforce the activity and make it more automated or habitual so that it becomes part of a person’s daily routine. |
| 27. | Use of follow up prompts | Use of reminders delivered after a person has started a behaviour change routine to help remind them to continue. |
| 28. | Facilitate social comparison | Individuals are encouraged to draw comparisons with others behaviour to increase motivation through modelling. |
| 29. | Plan social support | Prompt the person to elicit social support from other people and close relations to facilitate person’s successful completion of the behaviour. |
| 30. | Prompt identifcation as role model | The person views themselves as an example or role model to others for the behaviour. |
| 31. | Prompt anticipated regret | Induce expectations of shame, regret or guilt for failure to accomplish the goal. |
| 32. | Fear arousal | The presentation of fear-inducing information aimed at motivating change. |
| 33. | Prompt self-talk | The person is encouraged to talk to themselves before and during their activity to provide verbal encouragement and support. |
| 34. | Prompt use of imagery | The person is provided with instruction on how to use visualisation techniques and imagery to facilitate successful completion of the behaviour. |
| 35. | Relapse prevention | The person is encouraged to make plans to maintain behaviour that has been changed |
| 36. | Stress management | The person is encouraged to focus on reducing related stress and improving emotional control to reduce this as a barrier and promote oral hygiene. |
| 37. | Motivational interviewing | A clinical method including specific techniques to prompt changes by minimising resistance and resolving ambivalence to change. |
| 38. | Time management | Any technique that assists the person in managing their time efficiently, so as to be able to engage in the desired activity. For instance, effectively using a diary or organiser to plan time. |
| 39. | Communication skills training | Techniques directed at improving communication skills to improve interactions with others about the behaviour. |
| 40. | Stimulate anticipation of future rewards | Individuals are encouraged to consider future rewards associated with the outcome(s), without necessarily reinforcing behaviour change. |
